# Improved Accommodation for Inquests

**Published:** 1897-07-31

**Authors:** 


### IMPROVED ACCOMMODATION FOR INQUESTS.

We are very glad to hear that arrangements have now been made, or are approaching completion, throughout all the districts in London which will practically obviate the necessity for holding inquests any longer amid the peculiarly inappropriate surroundings of the public-house. By the co-operation of the various sanitary authorities with the joint committee which is charged with the provision of proper accommodation for the holding of inquests, good mortuary accommodation has been provided in nearly all the metropolitan districts. It is satisfactory also to learn that these mortuaries are increasingly used for the reception of bodies awaiting interment in other than inquest cases, and that the public are recognising the advantage of removing dead bodies, during the interval between death and interment, from crowded houses to the decent mortuaries which are being provided.

